# The Sanitary Commission

**Published:** 1864-02

**Authors:** 


### EDITORIAL AND MISCELLANEOUS.

*The Sanitary Commission.*—There is nothing more honorable to the age and the country in which we live than the voluntary associations for the relief of our soldiers in the field. The deaths from disease in the best provided armies in war have been to those from wounds as 9 to 1. (MacLeod's Surgical Notes of the Crimean War). If much of this mortality is caused by unavoidable evils, much is also due to unwholesome food, insufficient housing and clothing, discouragement and intemperance. In a mere pecuniary sense, it would be better for any government to take good care of its soldiers. "To preserve the soldiers," says Baudins, a most eminent military surgeon, "is the first interest of a nation which carries on a distant war, it is also the best security for final success."

This necessity our own government was, like others, incapable of providing for at the beginning of the war, but it was felt instinctively by the people whose charity found action through the various "Sanitary Commissions," and according to official statements, supplies to the value of about ten millions of dollars have passed through their hands. As this was in addition to all the government could do, it stands forth as a charity unrivalled in any age or nation. The British people made the same efforts for their soldiers in the Crimea to the extent of perhaps one-tenth the above amount, efforts which says a French surgeon, "show the real greatness of the nation.

It is impossible to estimate the benefits conferred in preserving life, soothing suffering, administering consolation to the sick and wounded, in correcting abuses in the hospitals and making medical officers more vigilant and attentive; in restoring our armies to the state of comparative health and vigor and efficiency which they now enjoy. It was charity following in the footsteps of relief to bind up his wounds.

Recently, when the enthusiasm seemed diminished and supplies were needed, it was proposed to hold a Fair of the Northwest at Chicago. Many thought it would be a failure, but the results far exceeded what could have been obtained at any former time. The Fair is thus described by the *Sanitary Commission Bulletin:*

The contributions to the Fair, to be sold for the benefit of our sick and wounded soldiers, were large, were munificent, but it was this tone of deep-seated earnestness which was largest. It was not merely what men and women said and did, but the *way* the thing was done, which carried with it this impression of wholesale generosity of spirit. Delicately wrought articles, such as usually adorn the tables of Fairs, the work of ladies' hands, were not wanting; but then the farmers from miles and miles around kept coming in with their wagons by twenties, and fifties, and hundreds, loaded down with their bulky farm produce; others came leading horses, or driving before them cows, or oxen, or mules, which they contributed instead of money, of which, perhaps, they had none; others brought live poultry which had been fed for months by the poor man's door; they brought this because they must bring something, and this was all they had. Some wagons were loaded from rich dairies, with butter and cheese by the ton. Then came great loads of hay from some distant farm, followed by others just as large from farms farther off. The mechanics brought their machines, and gave them in, one after another;—mowing machines, reapers, threshing machines, planters, pumps, fanning-mills—until a new building, a great storehouse, had to be erected to receive them; and here were ploughs, and stoves, and furnaces, and mill-stones, and nails by the hundred kegs, and wagons, and carriage springs,—and axes, and plate glass, and huge plates of wrought iron, (one the largest that was ever rolled from any rolling-mill in the world,) block tin and enameled leather, hides, boxes of stationery, and cases of boots, cologne by the barrel, native wine in casks, purified coal-oil by the thousand gallons—a mountain howitzer, a steel breech-loading cannon, a steam-engine made by the working-men in one of the manufactories of engines in Chicago—and on it this inscription—"This engine is donated by the workmen of the Eagle Works

Manufacturing Co., every man contributing something—not one Copperhead in the whole institution." There, too, were other machines which had been built by employees of various establishments, who had worked "after hours" to construct something for the Soldiers' Fair. Such, with a thousand other gifts, great and small, filled this new storehouse, where liberal purchasers were found waiting. Then, again, the carpenters, and joiners, who, in the press of work upon unfinished buildings, could not leave their hammer and saw to go to the Fair, joined together by tens and twenties, and set apart a day of which they would give their earnings to the soldiers. In like manner, different firms would advertise a sale for the benefit of the Fair. Thus,

"To the Loyal Stone-Masons of Chicago. We propose to donate to the Northwestern Sanitary Fair the entire proceeds of the sale of one canal boat load (20 tons) of our first-class rubble stone. Bids for the same will be received at our office till Friday next." Signed ———, &c.

Then loaded wagons came in long processions, toiling into the city, from far-off country places, bearing marks of frontier service, and the horses or mules, together with the drivers themselves, most of them told of wear. Many of them were sun-burnt men, with hard hands and rigid features; and a careless observer would have said that there was surely nothing in those wagons, as they passed, to awaken any sentiment. Yet something there was about it all which brought tears to the eyes of hundreds as the old farmers with their heavy loads toiled by.

Surgeon General Hammond has co-operated as have most of the volunteer surgeons and some of the regular army surgeons in this work, and the medical profession in general throughout the country has identified itself with it. The subject is not foreign to a medical journal. The spirit thus awakened may, when the war is ended, be turned to the relief of that class of sick poor, future soldiers and citizens, whose wants in this city and throughout the country are most inadequately provided for and who stand almost as much as the soldiers in need of medical and surgical care when disabled. To make known these wants, to aid in forming associations for their relief, in building hospitals, is the duty and legitimate office of the physician, for which he can in no better way prepare himself than by active aid to the "Sanitary Commission," and by which he can most effectually add to the usefulness and honor of the profession.

